# Sarpogrelate hydrochloride ameliorates diabetic nephropathy associated with inhibition of macrophage activity and inflammatory reaction in *db/db* mice

**DOI:** 10.1371/journal.pone.0179221

**Published:** 2017-06-22

**Authors:** Eun Soo Lee, Mi Young Lee, Mi-Hye Kwon, Hong Min Kim, Jeong Suk Kang, You Mi Kim, Eun Young Lee, Choon Hee Chung

**Affiliations:** 1 Department of Internal Medicine, Yonsei University Wonju College of Medicine, Wonju, Korea; 2 Department of Internal Medicine, Soonchunhyang University Cheonan Hospital, Cheonan, Korea; Faculty of Medicine & Health Science, UNITED ARAB EMIRATES

## Abstract

The aim of this study was to evaluate the effects of sarpogrelate hydrochloride (SH), a selective serotonin 2A receptor antagonist, on diabetic nephropathy in a type 2 diabetes mouse model. We treated *db/m* and *db/db* mice with SH (30 mg/kg/day) for 12 weeks. Rat renal proximal tubule cells (NRK-52E) and mouse macrophages (Raw 264.7) were stimulated by high glucose (30 mM glucose) or LPS (100 ng/ml) with or without SH (20 μM). We found that SH treatment increased serum adiponectin level and decreased urinary albumin, macrophage infiltration to glomeruli, and renal inflammatory and fibrosis signals, which were highly expressed in diabetic mice. Proximal tubule cells treated with high glucose (30 mM) also showed increased inflammatory and fibrosis signals. However, SH (20 μM) treatment reduced these changes. Moreover, SH treatment inhibited LPS-stimulated macrophage migration and activation. These findings suggest that SH ameliorates diabetic nephropathy not only by suppressing macrophage infiltration, but also by anti-inflammatory and anti-fibrotic effects.

## Introduction

Diabetic nephropathy (DN) is a progressive kidney disease that increases the morbidity and mortality of patients with diabetes globally. Several studies have shown that inflammatory cell accumulation in the kidney triggers renal inflammation, which is a key factor in the development and progression of DN [1,2]. Macrophages, one type of inflammatory cell, are known to mediate renal inflammation and fibrosis [3]. In the kidney, renal proximal tubular cells play an important role in the pathogenesis of DN. Inflammatory cells release mediators such as complements, antibodies, cytokines and chemokines, which activate proximal tubular cells and leading to the overproduction of matrix components causing renal fibrosis [4].

Serotonin (5-hydroxytryptamine, 5HT), a neurotransmitter released by activated platelets, acts on the brain and gastrointestinal tract. It has various functions and plays a role in regulating mood, urine storage, sleep, body temperature, food intake, and intestinal motility [5]. In addition, serotonin has powerful effects on vasoconstriction [6]. In diabetic patients, plasma serotonin level was elevated and associated with the development of cardiovascular complications [7]. Takahashi *et al*. showed that serotonin induced renal vasoconstriction in dog kidneys [8]. Moreover, serotonin increased mesangial cell proliferation, extracellular matrix synthesis, and fibrosis in diabetic nephropathy [9–11].

The action of serotonin is mediated by serotonin receptors, which are classified into seven subfamilies, from 5-HT1 to 5-HT7. One of these serotonin receptors, 5HT-2A, is found in mesangial cells, and serotonin modulates cytokine and chemokine production in lipopolysaccharide (LPS)-primed monocytes via serotonin receptors [12–14].

Sarpogrelate hydrochloride (SH), a selective 5HT-2A antagonist, is used clinically for the treatment of vascular inflammation and atherosclerosis [15]. A few studies have shown that SH mitigates albuminuria in diabetic nephropathy by hindering glomerular platelet activation [16,17]. However, the exact mechanisms underlying the role of SH in diabetic nephropathy are not fully understood. Thus, we investigated the effects and mechanisms of SH in diabetic nephropathy.

## Materials and methods

### Animal experiments

Male *db/m* and *db/db* mice in a C57BLKs/J background (6 weeks old) were purchased from Daehan Biolink (Chungbuk, Korea) and randomly divided into four groups (*n* = 7 in each group) as follows: 1) normal control (NC), 2) normal control treated with SH (NC+SH), 3) diabetic group (DB), and 4) diabetic group treated with SH (DB+SH). The SH (30 mg/kg/day) was administered via oral gavage for 12 weeks. Animals were housed at a constant temperature (20 ± 2°C) and humidity level (50–60%) with a 12-hour light and dark cycle with free access to water and food. Body weight and food intake were periodically measured, and urine was also periodically collected over 24 hours using a metabolic cage. After 12 weeks, animals were fasted for 8 hours and anesthetized with Zoletil (Virvac Laboratories, Carros, France) and xylazine hydrochloride (Rompun TS, Bayer AG, Leverkusen, Germany) by intraperitoneal injection. Blood samples were collected via intracardiac puncture and then centrifuged at 1,000 x g for 20 min to obtain serum. The serum was stored at -80℃ until use. After blood collection, the mice were perfused with PBS, and the kidney, perirenal fat, liver, and epididymal fat tissues were harvested. Part of each tissue was stored at -80℃ for analysis of mRNA and protein expression, and the other part was embedded with 4% paraformaldehyde for histological examination. All experiments were performed under the approval of the Institutional Animal Care and Use Committee (IACUC No. YWC-130430-1, Yonsei University, Wonju, Korea).

### Blood biochemistry

Serum glucose (Asan Pharmaceutical, Hwasung, Korea), total cholesterol (TC, Asan Pharmaceutical, Hwasung, Korea), triglycerides (TG, Asan Pharmaceutical, Hwasung, Korea), glutamate-oxaloacetate transaminase (GOT, Asan Pharmaceutical, Hwasung, Korea), glutamate-pyruvate transferase (GPT, Asan Pharmaceutical, Hwasung, Korea), insulin (Shibayagi Co., Shibukawa, Japan), and adiponectin (ADP, Adipogen, Seoul, Korea) levels were assessed using their corresponding commercial kits. HOMA-IR and HOMA-β were calculated from fasting glucose and insulin levels. HOMA-IR = Glucose (mg/dL) * Insulin / 405, HOMA-β = 360 * fasting insulin (μU/mL) / fasting glucose (mg/dL)– 63.

### Assessment of albuminuria

Urinary albumin (Exocell Nephrat II; Exocell Inc., Philadelphia, PA, USA) and creatinine (The Creatinine Companion; Exocell Inc.) levels in urine collected over 24 hours were measured according to the manufacturer’s instructions.

### Transmission electron microscopy

To evaluate ultrastructural changes in the glomeruli, extracted kidney tissues were fixed in 0.1 mol/L phosphate–buffered Karnovsky’s fixative, post-fixed in 1% phosphate-buffered osmium, and embedded in epoxy resin. Each specimen was thin-sectioned, and the number of slit pores and thickness of the glomerular basement membrane (GBM) were measured using a JEOL transmission electron microscope (JEM-1200EX II, JEOL Ltd., Tokyo, Japan). Electron micrographs of 10 glomeruli per kidney were randomly produced at 30K× for each mouse. Photomicrographs of the GBM were also analyzed to determine the density of slit pores between the podocyte foot processes. The number of slit pores was counted and divided by the GBM length (10 μm) to determine the linear density using an image analysis system (GmbH, SIS, Minster, Germany). The GBM thickness was assessed from measurements at three different cross-section sites

### Estimation of glomerular volume and fibrosis by light microscopy

Paraffin-embedded kidney tissues were cut into 4-μm-thick sections and stained with hematoxylin and eosin (H&E) and picrosirius red (Polysciences, Warrington, PA, USA) according to the manufacturer’s instructions. The stained sections were examined with an optical microscope that was equipped with a charge coupled device camera (Pulnix, Sunnyvale, CA, USA), and 10 glomerular areas per mouse were measured using an image analysis system (GmbH, SIS, Minster, Germany). In addition, the glomerular volume was calculated using the Weibel and Gomez formula: Glomerular volume (Gv) = Area 1.5 × 1.38/1.01 (1.38: shape coefficient, 1.01: size distribution coefficient) [18].

### Glomerular immunohistochemistry staining

The paraffin-embedded kidney tissue sections were analyzed for Sirt1, Cldn1, F4/80, and CD11c expression in the glomeruli. Areas positive for Sirt1, Cldn1, F4/80, and CD11c were measured in 10 glomerular areas per mouse at a magnification of 400× and analyzed using an image analysis system (SIS, GmbH, Munster, Germany).

### RNA extraction and quantitative real-time polymerase chain reaction

Total RNA from the kidney was isolated with TRIzol (Sigma) according to the standard protocol. cDNA was prepared from 1.0 μg of total RNA from each sample using a commercially available kit (QuantiTect reverse transcription kit; Promega, Hilden, Germany). Real-time PCR was used to measure the expression of NOS2 in the kidney. The NOS2 primers used were forward: 5’-TGGCCTCCCTCTGGAAAGA-3’, reverse: 5’-GGTGGTCCATGATGGTCACAT-3’. mRNA expression was assessed in an ABI PRISM 7900HT Sequence Detection System (Applied Biosystems) using the SYBR Green PCR master mix (Applied Biosystems, Foster City, CA, USA).

### Cell cultures

Rat renal proximal tubular epithelial cells, NRK52e cell line, were cultured at 37.5°C in Dulbecco's modified Eagle medium (DMEM) containing 5.5 mM glucose, 1% antibiotics, and 10% FBS. The cultured cells were fasted for 24 hours and treated with low-glucose (LG, 5.5 mM) and high-glucose media (HG, 30 mM) with or without SH (20 μM). Mouse macrophage cells (Raw264.7; ATCC) were cultured at 37.5°C in RPMI 1640 containing 1% antibiotics and heat inactivated 10% FBS. For the conditioned medium (CM), Raw 264.7 cells were stimulated by LPS (100 ng/ml) with or without SH (20 μM) for 24 hrs, and the collected CM was stored at -80℃ until use.

### Western blot analysis

Kidney tissues, NRK-52E cells, and Raw264.7 cells were homogenized in PRO-PREP protein extraction solution (iNtRON Biotechnology, Korea) with proteinase and phosphatase inhibitors (GenDEPOT, Barker, TX, USA). The concentration of supernatant protein was measured using the BCA protein assay kit (Pierce, Rockford, IL, USA). The protein samples were electrophoresed on sodium dodecyl sulfate-polyacrylamide gel electrophoresis (SDS-PAGE, 8–10%) gels and transferred to polyvinylidene difluoride membranes (PVDF). The blots were incubated with primary antibodies, including anti-Claudin1 (Cldn1), anti-NOS2, anti-PGC1ɑ, anti-Sirt1, anti-VEGF, anti-5-HT2A receptor (5-HT2AR) (Santa Cruz Biotechnology), anti-β-actin, anti-nephrin, anti-TGFβ, anti-TNFα (Abcam), anti-p-AMPK, anti-p-p38, anti-p38, anti-p-JNK, and anti-JNK, (Cell Signaling, Danvers, MA, USA) antibodies. Blots were visualized using a Biospectrum 600 imaging system (UVP, Upland, CA, USA).

### Migration assay

To determine whether LPS-mediated cell migration was inhibited by SH, we performed migration assays using 96-well chemotaxis chambers (Chemo Tx; Neuro Probe, Gaithersburg, MD, USA). The lower chambers were filled with 30 μl of media with or without LPS or SH, and macrophage cells were seeded (1.4 x 10^4^/50 μl) into the upper chambers and incubated for 4 hours at 37°C in 5% CO_2_. Migrating cells on the bottom side were fixed for 5 min in methanol, and their nuclei were stained with hematoxylin for 5 min. The cytoplasm was stained with eosin solution for 1 min, and the background was washed using tap water. Stained cells were then captured using an image analysis system (SIS, GmbH, Munster, Germany).

### Immunofluorescence staining in Raw264.7 cells

To determine 5-HT2AR expression changes in Raw 264.7 cells by SH, we performed immunofluorescence staining. Raw264.7 cells were seeded onto round coverslips (2 × 10^6^ cells) and stimulated with LPS with or without SH for 24 hours. After incubation, cells were fixed with 4% paraformaldehyde for 30 min at room temperature, blocked with 5% BSA for 1 hour at room temperature, then incubated with a primary rabbit anti-5-HT_2A_R antibody overnight at 4°C. For analyses, an Alexa Fluor 488-conjugated antibody was used as the secondary antibody. Coverslips were mounted with Fluoroshield and 4′6-diamidino-2-phenylindole (DAPI) (Sigma Chemical Corp., St. Louis, MO, USA) to label nuclei. A laser-scanning confocal microscope LSM 510 META (Carl Zeiss MicroImaging, Thornwood, NY, USA) was used to obtain the images.

### Statistics

Data are expressed as the mean ± SEM. Statistical analyses were conducted using SPSS version 18.0 for Windows to compare between the groups by student's t-test as applicable. P values < .05 were considered statistically significant.

## Results

### SH reduced body weight, epididymal fat and kidney weight gain

To evaluate the effect of SH administration on weight control, we periodically measured the body weight of mice. Over 12 weeks, the DB group gained more body weight compared to the NC group. The administration of SH to the DB group resulted in not only inhibited body weight gain and kidney weight gain but also inhibited epididymal fat gain and crown-like structures formation in epididymal fat compared to the DB group without SH. However, liver and kidney tissue weights were not significantly different compared to the DB group (Table 1 and S1 Fig). These results indicate that SH has the suppressing effect of kidney and fat weight gain while suppressing of the body weight gain.

**Table 1:** Influences of SH on physiological parameters in non-diabetic and diabetic mice.

		NC	NC+SH	DB	DB+SH
	Initial	1.7±0.2	20.8±0.4	35.6±0.6	34.9±0.2
Body weights (g)	Final	4.1±0.5	22±0.9	41.3±3.4	3.4±1.1
	Gain rate (%)	11.1	5.8	16^*^	0.9^+^
	EPD	0.17±0.01	0.12±0.02	1.03±0.05^*^	0.86±0.03^+^
Tissue weights (g)	Liver	1.03±0.03	1.06±0.05	2.78±0.11^*^	1.99±0.1
	Kidney	0.18±0.01	0.18±0.03	0.22±0.01^*^	0.2±0.01^+^

NC, normal mice group; NC+SH, normal mice with sarpogrelate hydrochloride treatment group; DB, diabetic mice group; DB+SH, diabetic mice with sarpogrelate hydrochloride treatment group; EPD fat, epididymal fat. Values shown are mean ± SEM.

*p < 0.05 vs. NC

†p < 0.05 vs. DB. *n* = 7 per group

### SH did not control blood glucose

After observing that SH affects body weight, we next analyzed fasting serum. Fasting serum glucose level was slightly decreased in the DB+SH group compared to the DB group (DB, 554.49 ± 47.72; DB+SH, 490.99 ± 49.85), although insulin levels were not different between the DB and DB+SH groups (DB, 0.491 ± 0.128; DB+SH, 0.321 ± 0.046). As a result, HOMA-insulin resistance (HOMA-IR) level was significantly increased in DB group compared to NC group but this level was not reduced by SH treatment. And HOMA-beta cell function (HOMA-β) levels were no significant differences among four groups (Table 2). These data indicate that SH administration does not control blood glucose.

**Table 2:** Comparison of biochemical characteristics of experimental animals.

	NC	NC+SH	DB	DB+SH
HOMA-IR	2.43±0.98	2.60±0.45	25.52±2.89^*^	21.58±3.94
HOMA-β	13.11±4.24	10.84±1.07	12.82±3.17	17.13±4.59
ADP (μg/ml)	2.99±0.12	2.73±0.32	1.16±0.07^*^	1.65±0.19^+^
TC (mg/dL)	27.14±2.48	33.31±7.12	106.2±15.26^*^	77.57±13
TG (mg/dL)	35.29±3.16	44.12±4.7	83.55±13.21^*^	73.32±3.21
GOT (IU/L)	19.61±2.51	19.13±2.14	43.79±19.14^*^	34.56±5.99
GPT (IU/L)	6.19±0.71	3.78±0.65	21.76±6.96^*^	11.48±2.33^+^

NC, normal mice group; NC+SH, normal mice with sarpogrelate hydrochloride treatment group; DB, diabetic mice group; DB+SH, diabetic mice with sarpogrelate hydrochloride treatment group; HOMA-IR, homeostasis model assessment-estimated insulin resistance; HOMA-β, homeostasis model assessment-estimated beta cell function; ADP, adiponectin; TC, total cholesterol; TG, triglycerides; GOT, glutamyl oxaloacetic transaminase; GPT, glutamyl pyruvic transaminase. Values shown are mean ± SEM.

*p < 0.05 vs. NC

†p < 0.05 vs. DB. *n* = 7 per group

### SH increased serum adiponectin

In this study, SH was shown to inhibit body weight and EPD fat gain. To further understand this lipid disorder, we measured serum TC and TG levels. Both TC and TG levels were significantly increased in the DB group, and although not significant, these levels were reduced after SH administration. Levels of the liver damage markers GOT and GPT were significantly increased in DB group compared to NC group, but SH administration to DB group slightly reduced GOT level and significantly reduced the GPT level. The adiponectin (ADP) level was significantly reduced in the DB group, while SH administration significantly increased the reduced ADP level (Table 2). These data indicate that SH improves the increased GPT level and reduced adiponectin levels in diabetic conditions.

### SH decreased albuminuria and recovered renal structures

**Fig 1:**
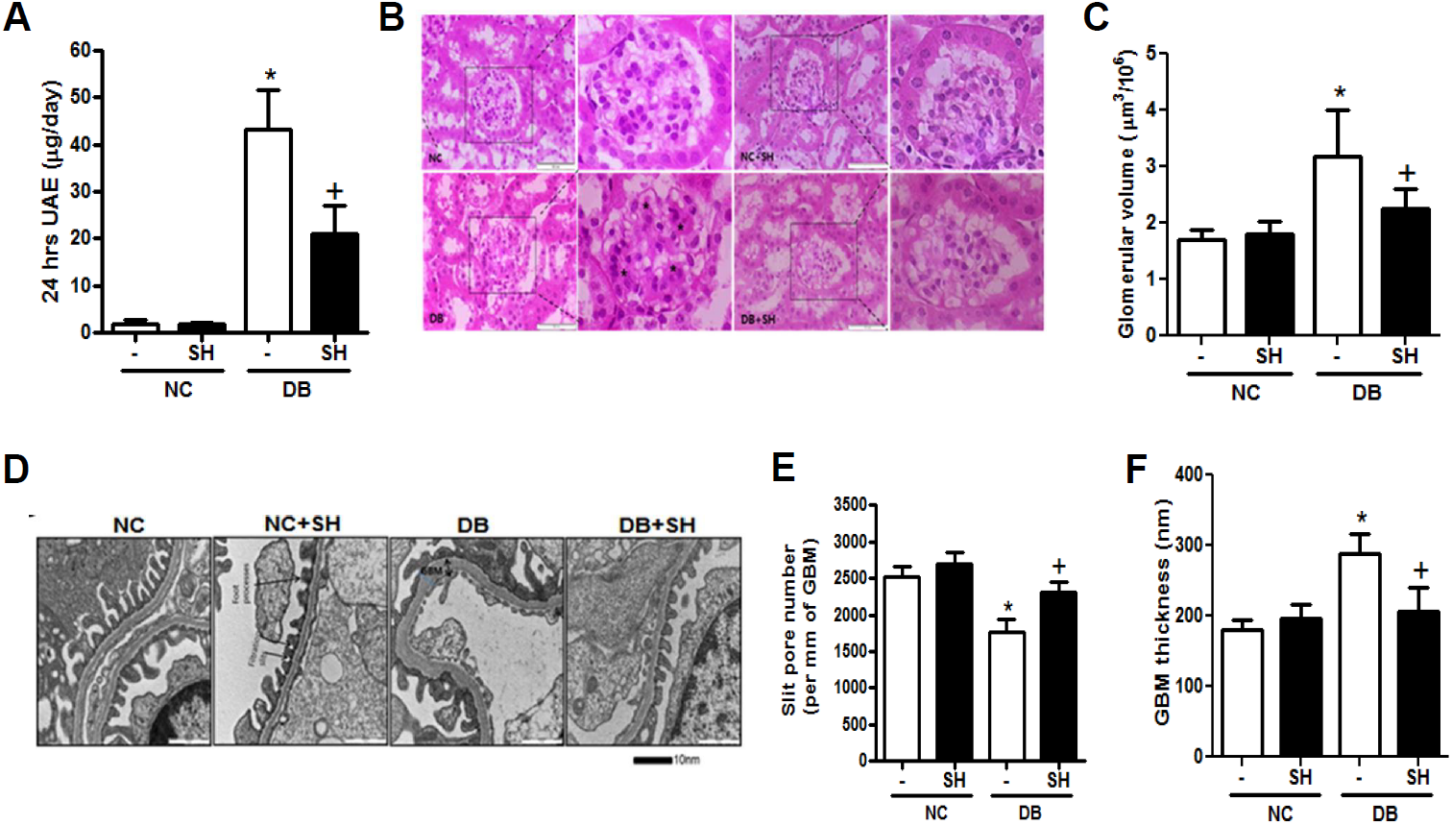
Effects of SH on the progression of diabetic nephropathy. After the 24 h urine collected by metabolic cages, albuminuria determined (A). Representative examples of glomerular hematoxylin and eosin (H&E)-stained sections (B). Differences of glomerular volume among four groups (C). Representative transmission electron microscope image of renal ultra stuctures (D). The differences of slit pore numbers (E) and glomerular basement membrane (GBM) thickness (F) among four groups. Original magnification: H&E staining, 400× and transmission electron microscopy, 30K×. NC, normal mice group; NC+SH, normal mice with sarpogrelate hydrochloride treatment group; DB, diabetic mice group; DB+SH, diabetic mice with sarpogrelate hydrochloride treatment group; UAE, urine albumin excretion. Values shown are mean ± SEM. ^*^p < 0.05 vs. NC, ^†^*p* < 0.05 vs. DB. *n* = 7 per group.

Mouse models of obese type 2 diabetes including the *db/db* mouse are found to have albuminuria compared to control mice. At 20 weeks of age, 24-hour urine albumin excretion was exceedingly increased in the DB group, but was significantly decreased by SH administration (Fig 1A). Histopathological changes in DN include thickening of the GBM mesangial expansion and podocyte effacement [19]. In this study, mesangial cell expansion and glomerular hypertrophy were observed in the DB group, both of which decreased after SH administration for 12 weeks (Fig 1B and 1C). In addition, increased GBM thickness and podocyte effacement as well as decreased slit pore density were recovered through the administration of SH (Fig 1D–1F). These data indicate that SH administration reduced albuminuria by recovering the damage and reversing changes to renal structures.

### SH recovered DB-induced renal molecular changes

**Fig 2:**
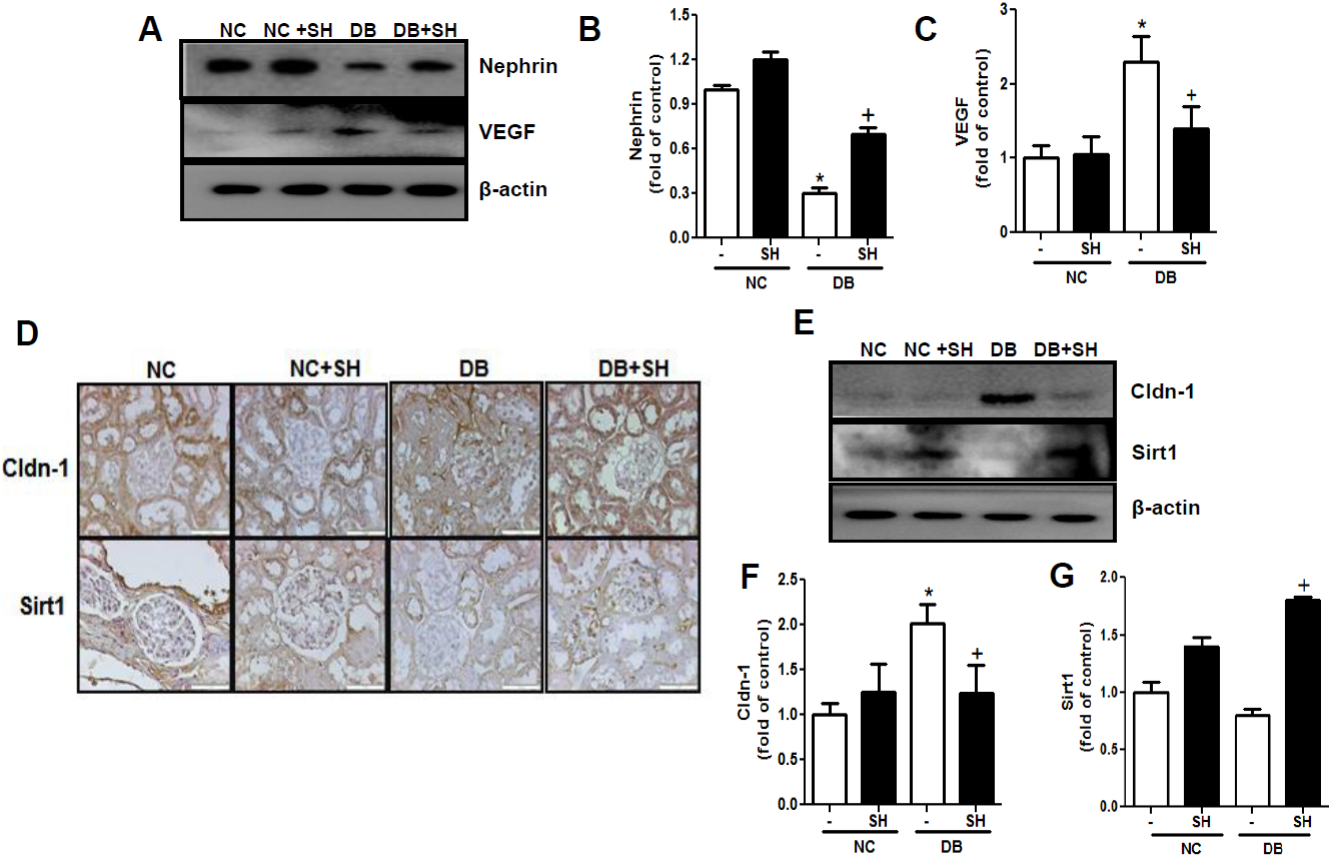
Western blot analysis of the renal cortex after treatment with sarpogrelate hydrochloride. The renal expression of nephrin and VEGF was analyzed by Western blotting (A). The relative expression of nephrin (B) and VEGF (C) were analyzed by ImageJ software. The expression of renal Cldn1 and Sirt1 was analyzed by IHC (D) and western blotting (E).The difference renal nephrin and VEGF levels among four groups. NC, normal mice group; NC+SH, normal mice with sarpogrelate hydrochloride treatment group; DB, diabetic mice group; DB+SH, diabetic mice with sarpogrelate hydrochloride treatment group. Values shown are mean ± SEM. ^*^p < 0.05 vs. NC, ^†^*p* < 0.05 vs. DB. *n* = 6 per group.

Renal nephrin is located at the slit diaphragm of the glomerular podocyte for proper functioning of the renal filtration barrier [20]. Overexpressed VEGF has been found to positively correlate with proteinuria [21]. SH administration to the DB group significantly recovered the decreased renal nephrin (Fig 2A and 2B) and the increased VEGF level (Fig 2A and 2C). In the kidney, Sirt1-Cladn1 crosstalk regulates renal function by inhibiting expression of the tight junction protein Cldn1 by Sirt1. In this study, renal Cldn-1 expression was significantly increased, while Sirt1 expression was decreased in the DB group compared to the NC group. These altered expressions were significantly recovered by SH administration (Fig 2D, 2E and 2F). These findings suggest that SH administration recovers renal damage induced by changes in the expression of proteins such as decreased renal nephrin and Sirt1 and increased VEGF and Cldn-1.

### SH inhibited macrophage infiltration and expression of NOS2 and TNFα

**Fig 3:**
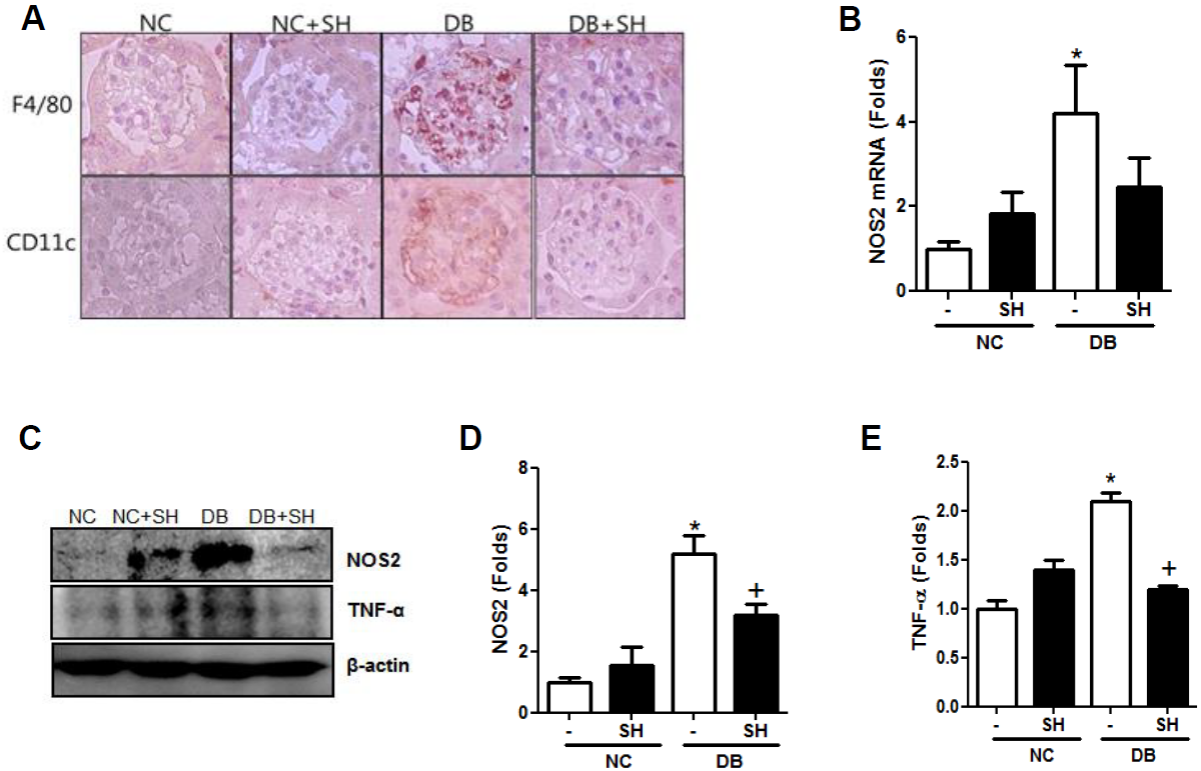
SH modulated inflammatory-macrophage accumulation and inflammatory response. The levels of mouse mature macrophage marker F4/80 and inflammatory macrophage marker CD11c were staining of kidney sections (A). The changes of NOS mRNA (B) and protein expression (C and D) in kidney. And the inflammatory cytokines, TNF-α was analyzed by western blotting (C and E). NC, normal mice group; NC+SH, normal mice with sarpogrelate hydrochloride treatment group; DB, diabetic mice group; DB+SH, diabetic mice with sarpogrelate hydrochloride treatment group. Values shown are mean ± SEM. ^*^p < 0.05 vs. NC, ^†^*p* < 0.05 vs. DB. *n* = 6 per group.

Macrophage infiltration in glomeruli was evaluated via macrophage staining. Accumulation of F4/80- and CD11c (M1 pro-inflammatory)-positive cells was greatly increased in the DB group, but these accumulation patterns were decreased by SH administration (Fig 3A). The inflammation-associated enzyme NOS2 plays a central role in the inflammatory reaction. The increased renal NOS2 mRNA and protein levels in the DB group were decreased after SH administration (Fig 3B, 3C and 3D). Moreover, expression of the inflammatory cytokine TNF-α also increased in the DB group compared to the NC group and was decreased by SH administration (Fig 3C and 3E). Collectively, these data indicate that SH inhibits macrophage infiltration to glomeruli and inflammation.

### SH decreased inflammatory signals in rat renal proximal tubule cells

**Fig 4:**
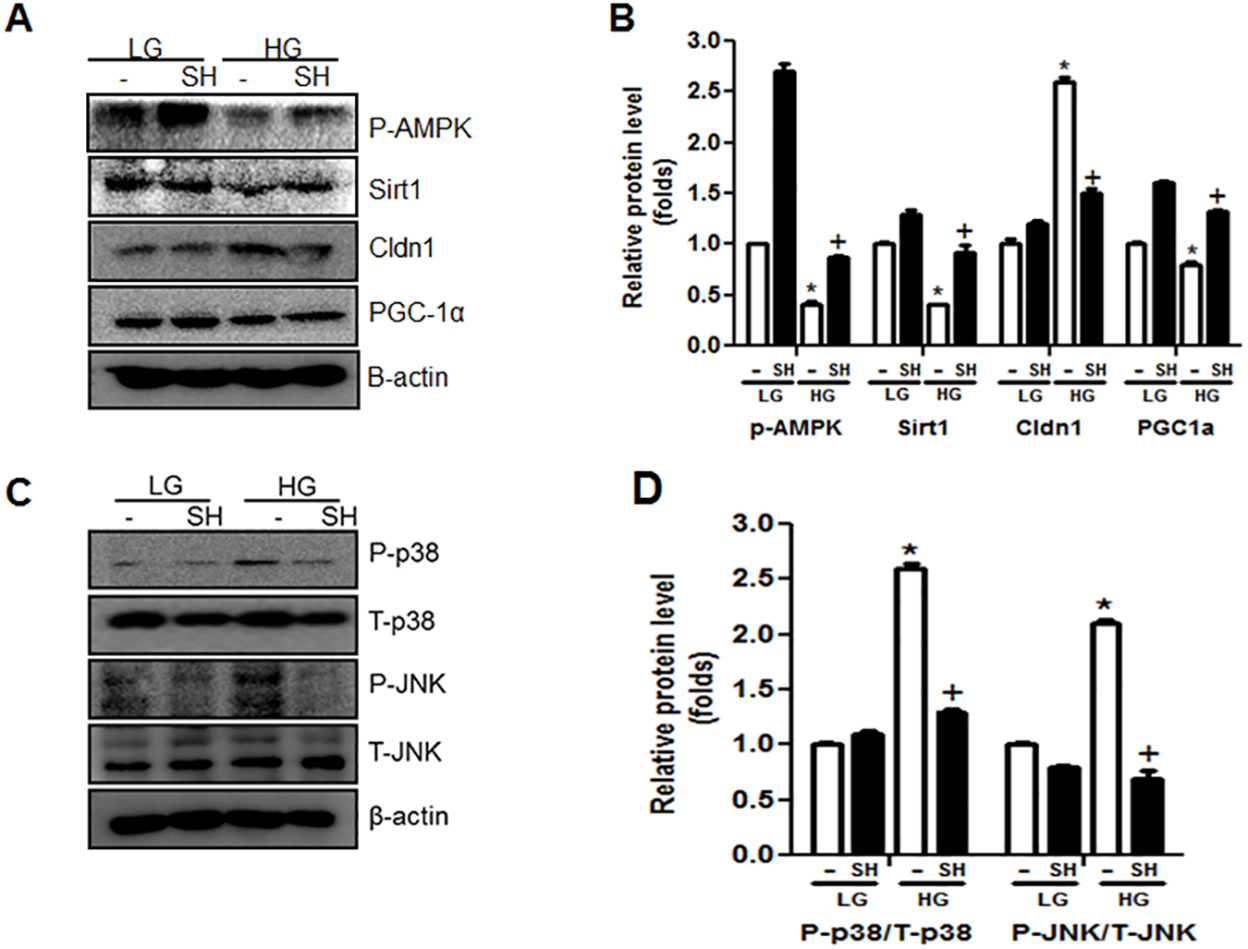
SH inhibition of NRK52E cell glucotoxicity depends on the AMPK-SIRT1-PGC1alpha axis and inflammatory signals. NRK52E cells were stimulated by HG with or without SH and analyzed of phosphorylated AMPK, Sirt1, PGC1α and Cldn1 expressions (A and B). Andinflammatory molecules such as phosphorylated p38 and phosphorylated JNK changes were analyzed in HG with or without SH cultured NRK52E cells (C and D). LG, 5.5 mM D-glucose; HG, 30 mM D-glucose; SH, sarpogrelate hydrochloride. Values shown are mean ± SEM. ^*^p < 0.05 vs. LG, ^†^*p* < 0.05 vs. HG.

High glucose treatment to NRK-52E cells reduced the expression of p-AMPK, Sirt1, and PGC-1α, and SH treatment recovered these changes. Interestingly, SH treatment also restored the expression of Cldn1 to normal level. The Cldn1 level was increased by HG and significantly decreased by SH treatment (Fig 4A and 4B). SH also inhibited inflammatory signals such as the phosphorylation of p38 and JNK. Phospho-p38 and phospho-JNK levels were increased by HG stimulation in NRK-52E cells, and these levels were decreased by SH treatment (Fig 4C and 4D). These data indicate that SH not only increases AMPK-Sirt1-PGC1α pathways, but also inhibits HG-induced inflammatory signals in renal proximal tubule cells.

### SH decreased activation and cell migration of macrophages in Raw264.7 cells

**Fig 5:**
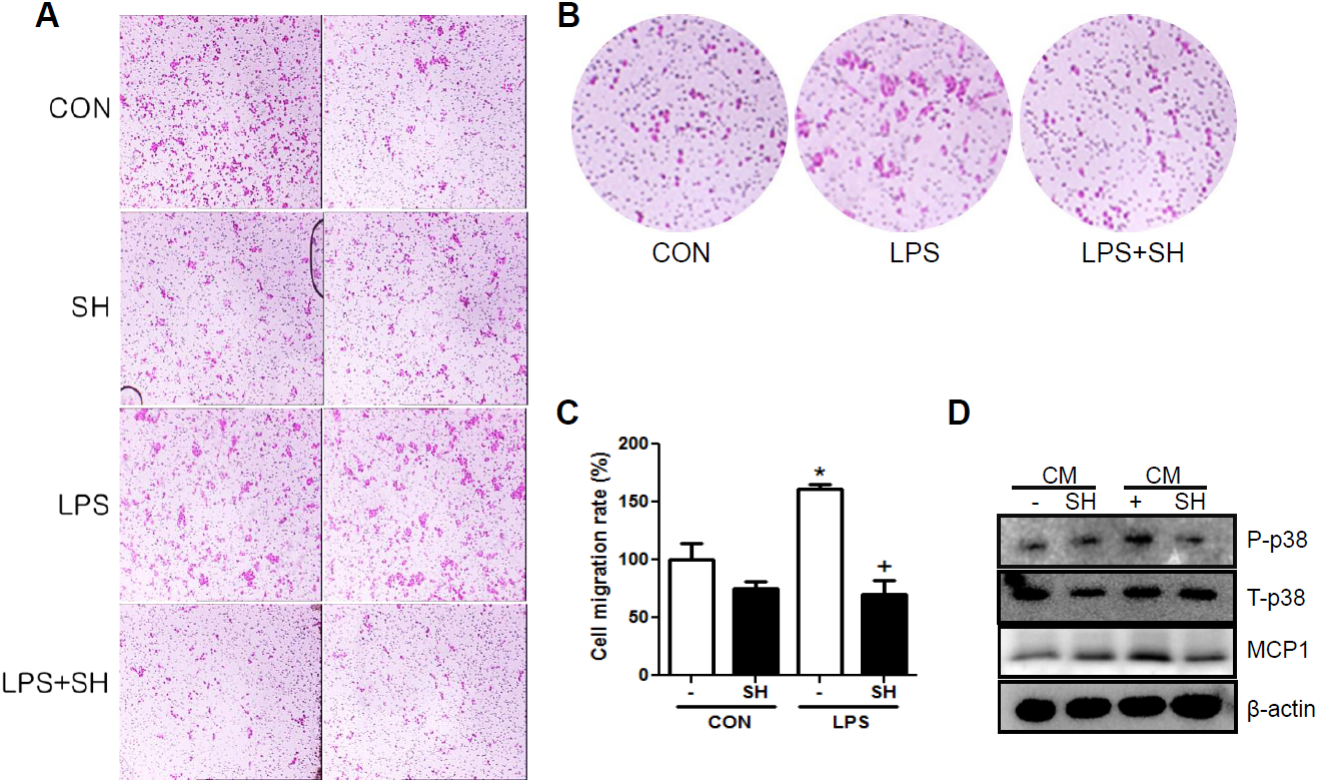
SH reduces macrophage migration and activation. The differences of cell migration rate (A and C) and morphological change (B) by LPS with or without SH in Raw264.7 cells. (B) Change of phosphorylated p38 and MCP1 expressions by SH treatment in macrophage cultured media stimulated NRK52E cells (D). CON, control; LPS, lipopolysaccharides; SH, sarpogrelate hydrochloride; CM, conditioned medium. Values shown are mean ± SEM. ^*^p < 0.05 vs. CON, ^†^*p* < 0.05 vs. LPS.

Renal infiltration of macrophages induces inflammatory cytokines and activates inflammatory signals. LPS stimulation increased macrophage migration and showed morphological changes indicating macrophage activation. However, SH treatment inhibited both cell migration and morphological changes (Fig 5A, 5B and 5C). Moreover, when macrophage cultured media with LPS was transferred to renal proximal tubule cells, the levels of both the inflammatory cytokine MCP1 and inflammatory signaling molecule phospho-p38 were increased. SH treatment with CM reduced the increases in MCP1 and phospho-p38 levels. These findings suggest that SH not only inhibits LPS-induced macrophage activation and cell migration in mouse macrophages, but also Raw264.7-induced inflammation in renal proximal tubule cells.

### SH reduced 5-HT2A receptor expression in macrophage cells

**Fig 6:**
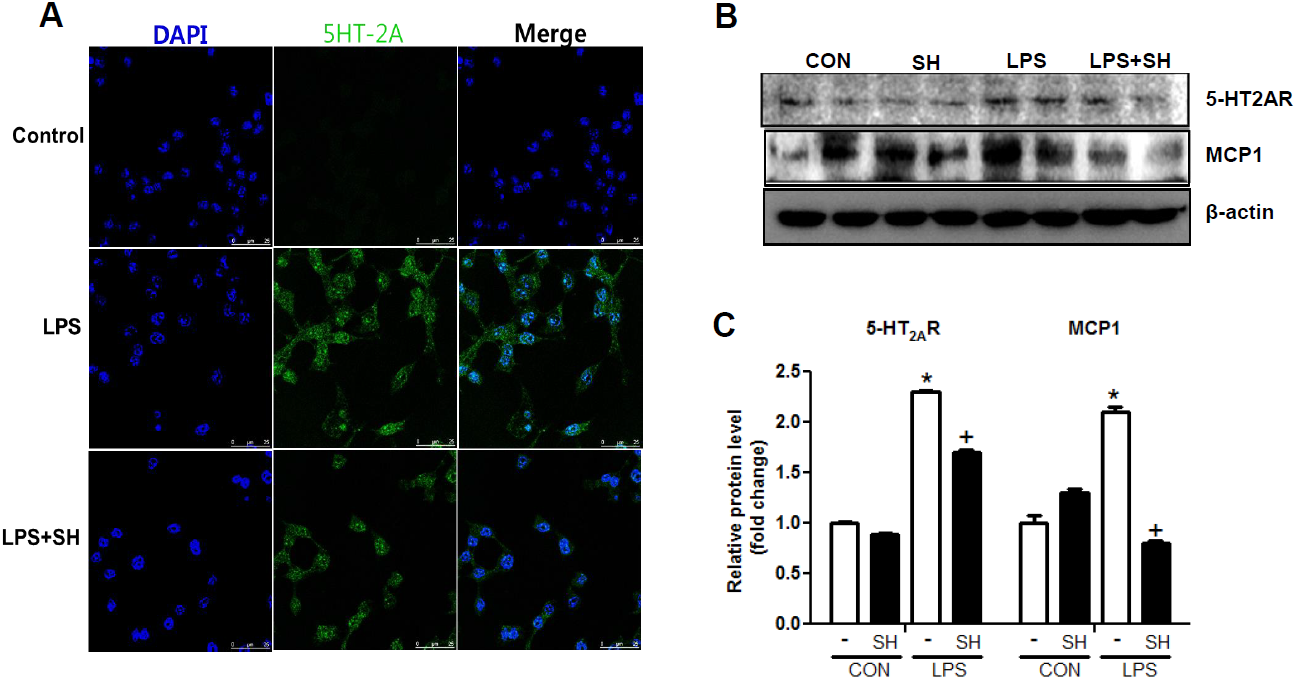
SH inhibits the 5HT-2A receptor and MCP1 in RAW264.7 cells. Representative immunofluorescence staining of 5HT-2A receptor (A) and western blotting of 5HT-2A receptor and MCP1 levels (B and C)in Raw264.7 cells. CON, control; LPS, lipopolysaccharides; SH, sarpogrelate hydrochloride. Values shown are mean ± SEM. ^*^p < 0.05 vs. CON, ^†^*p* < 0.05 vs. LPS.

To evaluate the receptor-inhibiting function of SH, we stained 5HT-2A in Raw 264.7 cells. LPS stimulation increased the expression of 5-HT2AR. However, the increased 5-HT2AR expression was significantly inhibited by SH treatment (Fig 6A, 6B and 6C). Moreover, LPS-induced inflammatory cytokine MCP1 was significantly decreased by SH treatment (Fig 6B and 6C). These results indicate that SH, a selective 5HT-2A receptor antagonist inhibits LPS-induced 5HT-2A receptor expression and MCP-1.

### Sarpogrelate hydrochloride inhibited renal fibrosis

**Fig 7:**
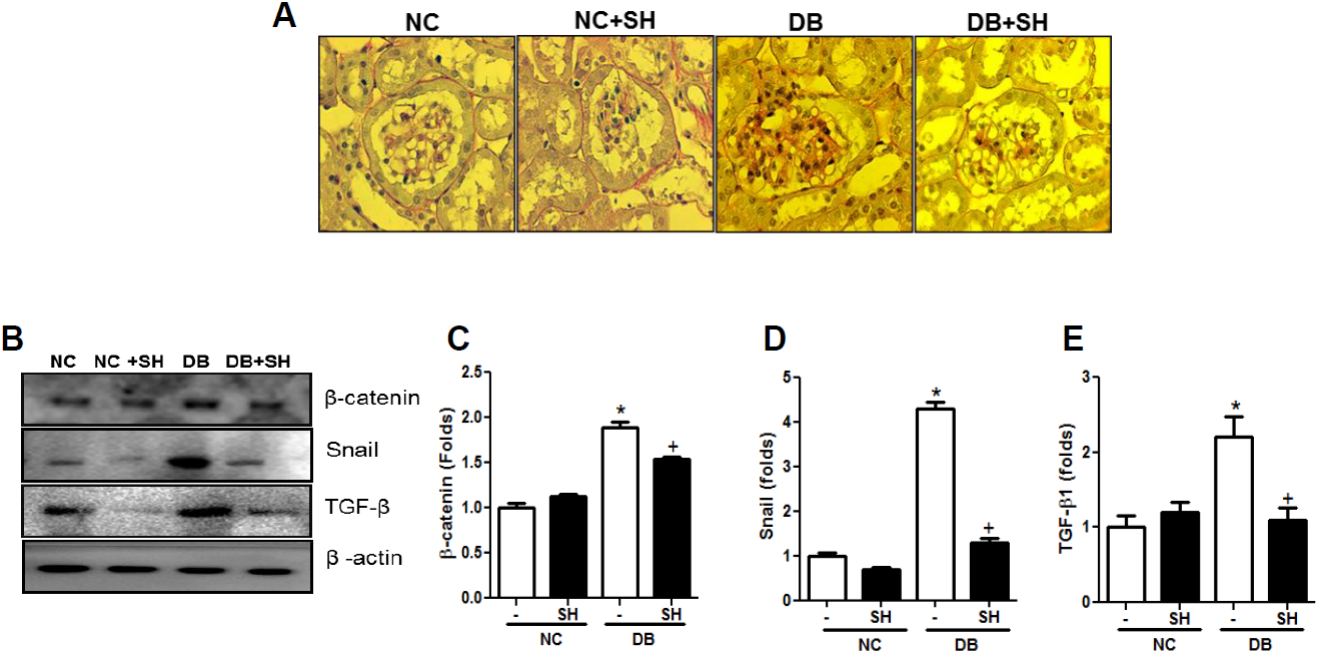
SH ameliorates renal fibrosis in DN. Collagen accumulation in glomeruli was analyzed by picrosirius red staining in the kidney sections (A) and fibrosis-related molecules such as β-catenin, snail, and TGF-β (B, C, D and E) were analyzed by western blotting. NC, normal mice group; NC+SH, normal mice with sarpogrelate hydrochloride treatment group; DB, diabetic mice group; DB+SH, diabetic mice with sarpogrelate hydrochloride treatment group. Values shown are mean ± SEM. ^*^p < 0.05 vs. NC, ^†^*p* < 0.05 vs. DB. *n* = 6 per group.

Picrosirius red staining was used to identify the presence of collagen. In this study, collagen accumulation in glomeruli was increased in the DB group compared to the NC group, but SH reduced collagen accumulation (Fig 7A). Increases in β-catenin, snail, and TGF-β1 were also decreased in the SH+DB group compared to the DB group (Fig 7B). These results indicate that SH has both anti-inflammatory and anti-fibrotic effects.

## Discussion

In diabetes, macrophage-mediated chronic inflammation is a major risk factor of diabetes-associated complications including DN [22]. Inflammatory cells such as macrophages and T cells accumulate in the renal glomeruli and interstitium in the early stages of diabetic nephropathy, and these activated macrophages have pro-inflammatory, pro-fibrotic, and angiogenic effects [23,24]. In our previous study, we demonstrated that macrophages strongly correlated with the progression of DN. C-C chemokines have an important role in the recruitment of inflammatory cells to the kidney through their receptors. One of the key chemokines, monocyte chemoattractant protein-1 (MCP-1), regulates the migration and infiltration of monocytes and macrophages through its receptor CCR2. Treatment with a CCR2 inhibitor ameliorated DN due to the inhibition of macrophage infiltration to glomeruli and inflammation [25].

Serotonin is a neurotransmitter that is synthesized from the amino acid L-tryptophan in a metabolic pathway involving two major enzymes, TPH1 and THP2. In normal human, serotonin contained in the blood approximately 10 pM in plasma, 1 μM in serum, and 400 μM in platelets [26]. Once serotonin is released, it is rapidly taken up and stored in lymphocytes, monocytes, and macrophages through the seven types of receptors (5-HT1 to 5-HT7) and increases monocyte adhesion and macrophage foam cell formation [27]. In this study, 5-HT2A expression was increased in macrophage cultured with serum contained media compared to serum free media. Not only that, HG or LPS stimulation also increased 5-HT2A expression in macrophages and renal proximal tubule cells (S2 Fig). Monocytes migrate across the endothelium along the chemotactic gradient into tissues and then differentiate into macrophages [28]. The specific stimulation of 5-HT receptors such as 5-HT1 and 5-HT2 activates NF-κB and MAPKs in many cell types including immune cells and leads to inflammatory responses [29]. Innate immune cells are the major sources of inflammatory diseases such as dextran sulfate sodium-induced colitis [30].

Ketanserin, a selective 5-HT2AR antagonist used for treatment in colitis model mice, alleviates colitis through its anti-inflammatory effects [31]. Moreover, SH is also a serotonin 2A receptor antagonist and has been used clinically for cutaneous ulcers and ischemic changes resulting from arteriosclerosis [32]. Interestingly, several studies have reported that SH had renoprotective effects in DN by reducing glomerular platelet activation and increasing serum adiponectin level [17,33]. However, the exact mechanisms underlying the beneficial effects of SH in DN are not clearly understood.

In type 2 diabetes, hyperglycemia is a crucial factor in the development of DN due to increases in glycation, oxidative stress, glomerular hyperfiltration, hypertension, and activation of the polyol pathway and protein kinase C. These changes induce inflammatory pathways and increase circulating inflammatory cells [34]. In this study, expression of the inflammatory macrophage markers F4/80 and CD11c was found in glomeruli. These markers were noticeably detected in the glomeruli of the DB group compared to the NC group, and the administration of SH inhibited this increase. Moreover, SH administration not only inhibited macrophage infiltration, but also reduced inflammatory mediators such as TNF-α and NOS2. LPS plays an important role in the pathogenesis of inflammation. In addition to serum glucose, the serum LPS level also significantly increased in diabetic patients compared to non-diabetic patients, and the increased serum LPS activity was strongly associated with the components of metabolic syndrome and development of microalbuminuria [35,36]. LPS produced prostaglandins such as PGD_2_ and PGE_2_ concomitant to eliciting macrophage migration [37]. To demonstrate the effects of SH on macrophage migration, the murine macrophage cell line Raw 264.7 was stimulated by LPS. LPS stimulation increased both macrophage cell activation and cell migration, which were inhibited by SH treatment.

Next, we examined the effects of SH on LPS-stimulated macrophages on normal renal proximal tubule cells. The transfer of LPS-stimulated macrophage cultured media to renal proximal tubule cells increased the levels of MCP-1 and phospho p-38 MAPK, while SH treatment decreased these changes.

The glucose lowering effects of SH have also been examined in previous studies [38,39]. SH inhibited glucose-induced insulin release from the pancreas and increased glucose uptake in C2C12 myoblast cells [39]. However, in this study, the fasting blood glucose level was decreased but not significantly after SH administration. In addition, the increased fasting insulin level in the DB group did not change after SH administration. Similar to our experiment, SH administration did not change the fasting blood glucose level in HFD/STZ-induced nephropathy mice [40]. Therefore, additional studies should be performed to understand the mechanism of action of SH in diverse animal models. However, serum adiponectin level was noticeably recovered by SH in *db/db* mice.

The adipocyte-derived cytokine adiponectin is known to regulate macrophage polarization into the anti-inflammatory phenotype, consequently blunting the progression of metabolic and cardiovascular disease [41,42]. The plasma adiponectin concentration in diabetic patients was lower than in normal subjects, and SH administration in type 2 diabetic patients increased circulating adiponectin [42,43]. Also, adiponectin has been shown to increase insulin sensitivity and fatty acid oxidation and decrease muscle lipid content, inflammation, and vascular injury [44]. In a type 2 diabetic rat model, SH treatment reduced TG and free fatty acids and improved insulin resistance [38]. Fatty acid oxidation was increased by adiponectin via activation of the AMPK signaling pathway [45], while AMPK inhibited fatty acid-induced inflammation by increasing Sirt1 expression [46]. Several studies have shown that target reagents for the treatment of DN had a therapeutic effect through AMPK activation [47–49]. Sirt1 is a NAD^+^-dependent deacetylase that mediates the effects of caloric restriction, stimulates AMPK, and improves mitochondrial function, and its dysfunction is associated with metabolic diseases [50]. AMPK/Sirt1 activation reduced obesity-associated macrophage infiltration and inflammation in adipose tissue [51]. Myeloid-specific deletion of the Sirt1 gene promotes macrophage infiltration into insulin-sensitive organs and exacerbates tissue inflammation [52]. In DN, Sirt1 expression was lower in both the proximal tubules and glomeruli, while Cldn1 expression was higher in the kidneys of diabetic patients. Cldn1 activated the β-catenin–snail pathway, which induces glomerular barrier damage through the down-regulation of synaptopodin or podocin in podocytes and leads to EMT in renal proximal tubule cells [53,54]. Renal proximal tubule-specific SIRT1 overexpression reduced Cldn1 expression in glomeruli and attenuated DN in *db/db* mice [53]. In our study, renal p-AMPK and Sirt1 levels were decreased, while the Cldn1 level was increased in the diabetic group. Additionally, HG stimulation also decreased Sirt1 and increased Cldn1, while SH treatment reversed these changes in renal proximal tubule cells.

Collectively, these results indicate that macrophage infiltration into the kidney is necessary for renal injury in diabetes, and SH administration increased adiponectin and inhibited macrophage infiltration. We also showed that SH has an anti-inflammatory effect through its inhibition of macrophage activity and inflammatory reaction in *db/db* mice.

In conclusion, this study suggests that SH could be a therapeutic agent in patients with diabetic nephropathy.

## Supporting information

S1 Fig Macrophage infiltration in adipose tissues. Representative H&E staining of epididymal fat. NC, normal mice group; NC+SH, normal mice with sarpogrelate hydrochloride treatment group; DB, diabetic mice group; DB+SH, diabetic mice with sarpogrelate hydrochloride treatment group.
(TIF)

S2 Fig 5HT-2A expression in Raw264.7 and NRK52E cells. The changes of 5HT-2A after LPS time-dependently treatment in Raw264.7 cells (A). The differences of 5HT-2A expression after cultured with serum free media or FBS contained media. And the change of 5HT-2A was analyzed by western blotting after LPS treatment with or without SH in Raw164.7 cells (B). The changes of 5HT-2A by SH in HG or LPS stimulated NRK-52E cells (C).
(TIF)

S1 File Dataset. (XLS)

S2 File Figure. (PDF)
