# Supplementary figures and images for: Sarpogrelate hydrochloride ameliorates diabetic nephropathy associated with inhibition of macrophage activity and inflammatory reaction in db/db mice
Source: PLoS One. 2017 Jun 22;12(6):e0179221. doi: 10.1371/journal.pone.0179221 (PMC5480859; doi:10.1371/journal.pone.0179221)

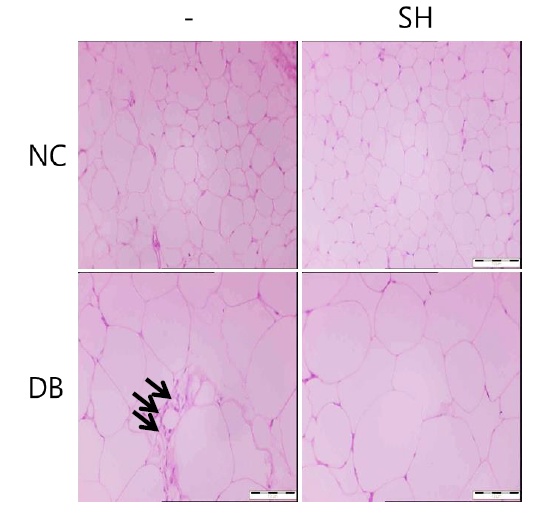

Supplement: S1 Fig — Representative H&E staining of epididymal fat. NC, normal mice group; NC+SH, normal mice with sarpogrelate hydrochloride treatment group; DB, diabetic mice group; DB+SH, diabetic mice with sarpogrelate hydrochloride treatment group. (TIF) [file pone.0179221.s001.tif]

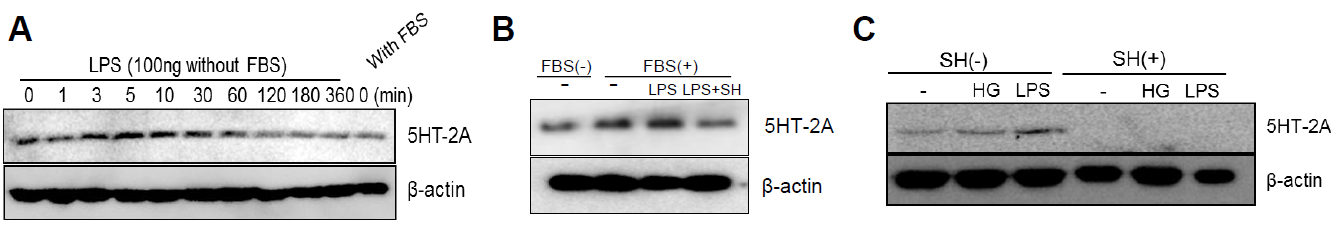

Supplement: S2 Fig — The changes of 5HT-2A after LPS time-dependently treatment in Raw264.7 cells (A). The differences of 5HT-2A expression after cultured with serum free media or FBS contained media. And the change of 5HT-2A was analyzed by western blotting after LPS treatment with or without SH in Raw164.7 cells (B). The changes of 5HT-2A by SH in HG or LPS stimulated NRK-52E cells (C). (TIF) [file pone.0179221.s002.tif]
