# Supplementary material for: Sarpogrelate hydrochloride ameliorates diabetic nephropathy associated with inhibition of macrophage activity and inflammatory reaction in db/db mice
Source: PLoS One. 2017 Jun 22;12(6):e0179221. doi: 10.1371/journal.pone.0179221 (PMC5480859; doi:10.1371/journal.pone.0179221)
Supplement: S2 File — (PDF) [file pone.0179221.s004.pdf]

Fibrosis staining (Picroscirious red staining, magnification: x400)

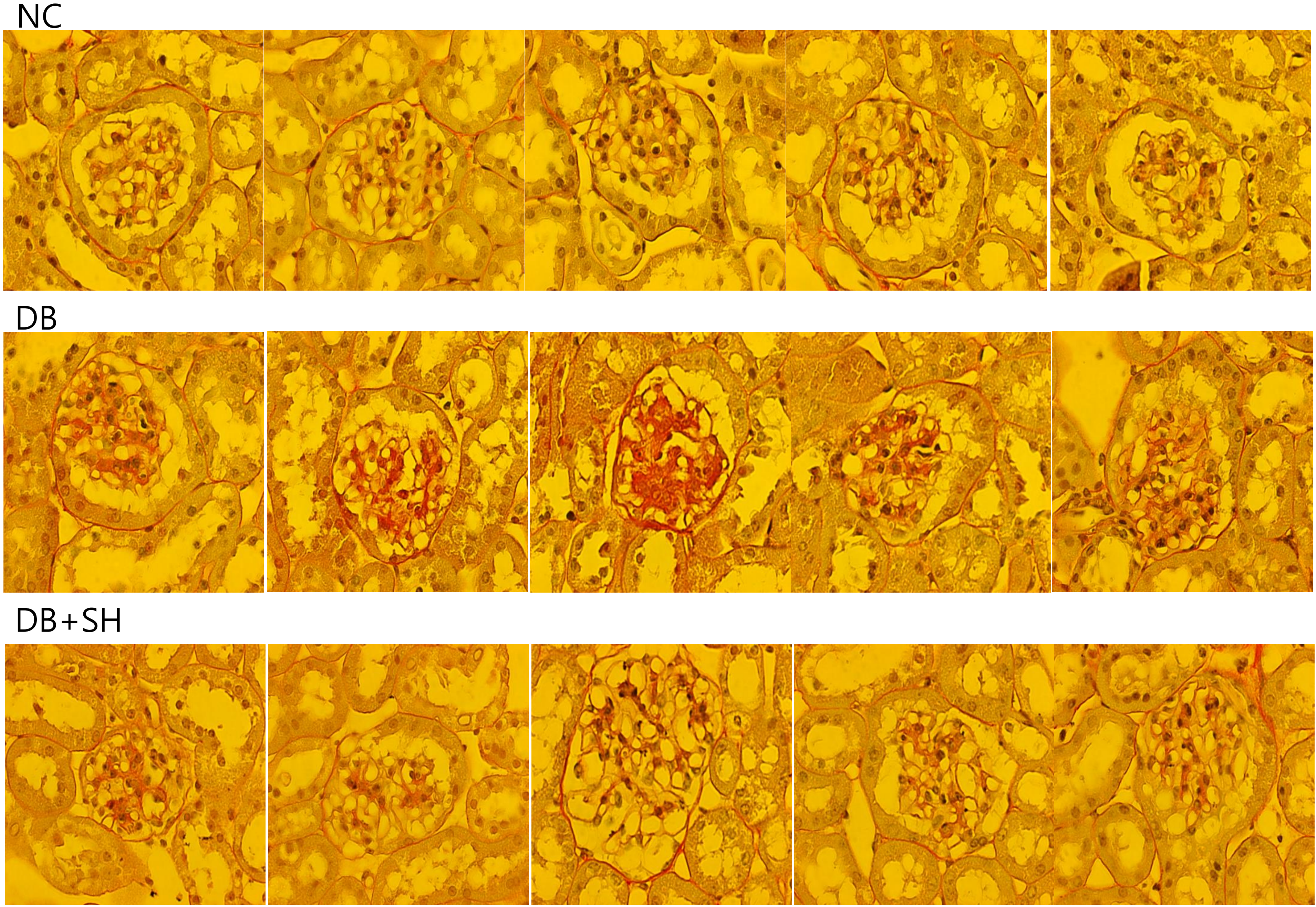

H&E staining (magnification, x 400)

NC

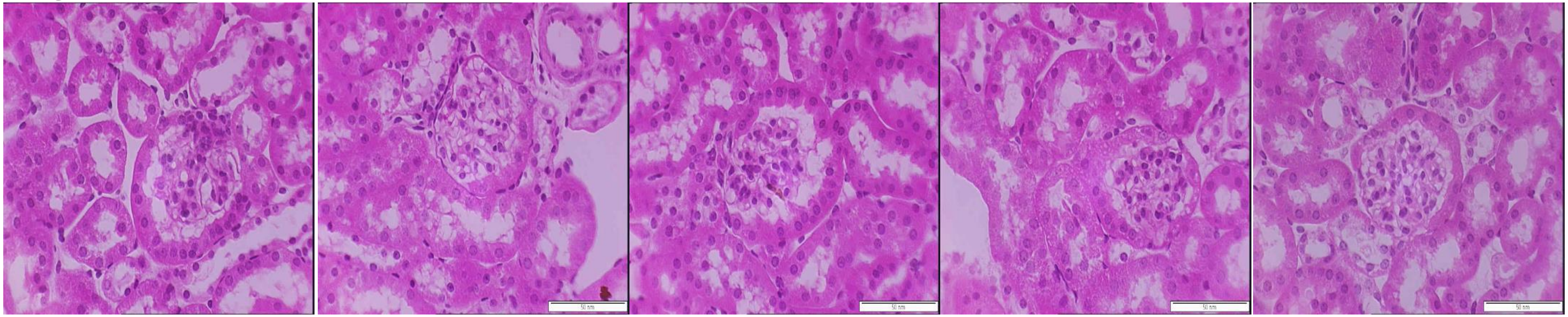

DB

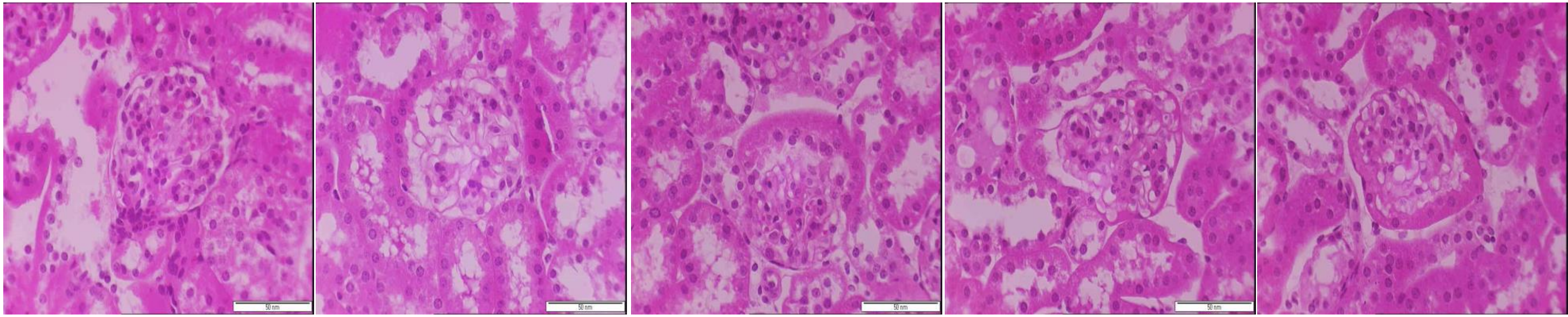

DB+SH

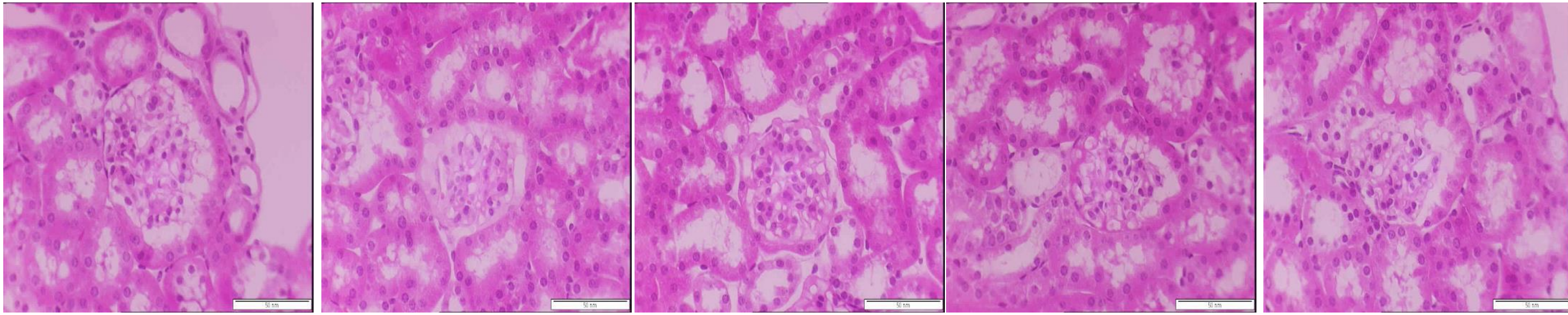

EM (magnification, x 30K)

NC

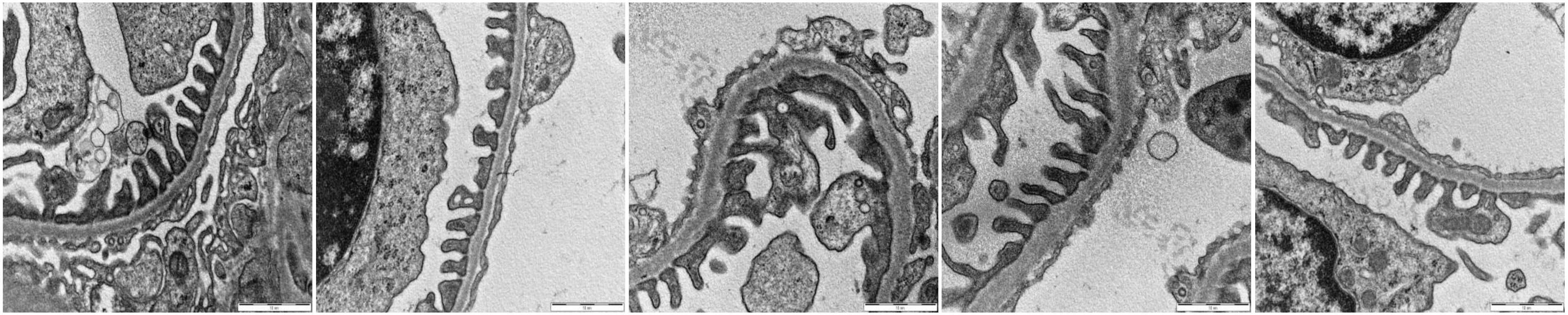

DB

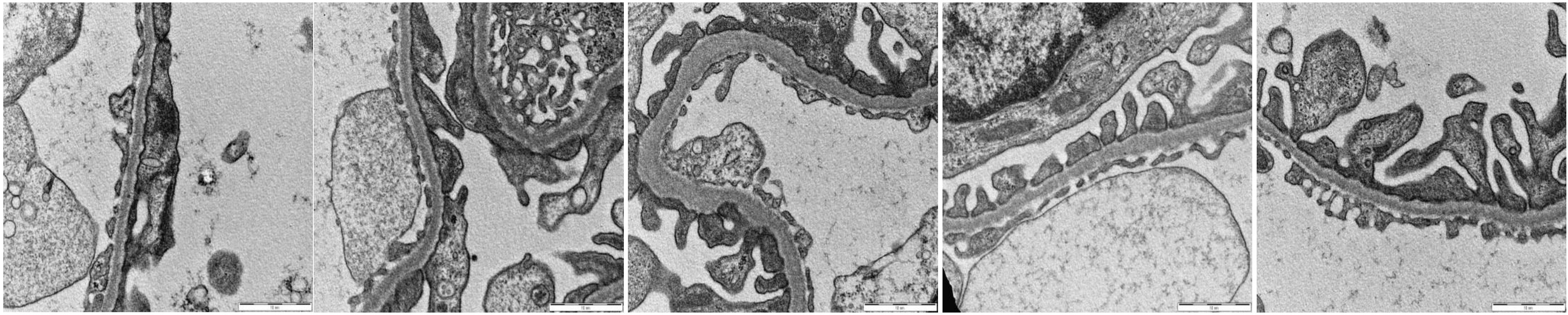

DB+SH

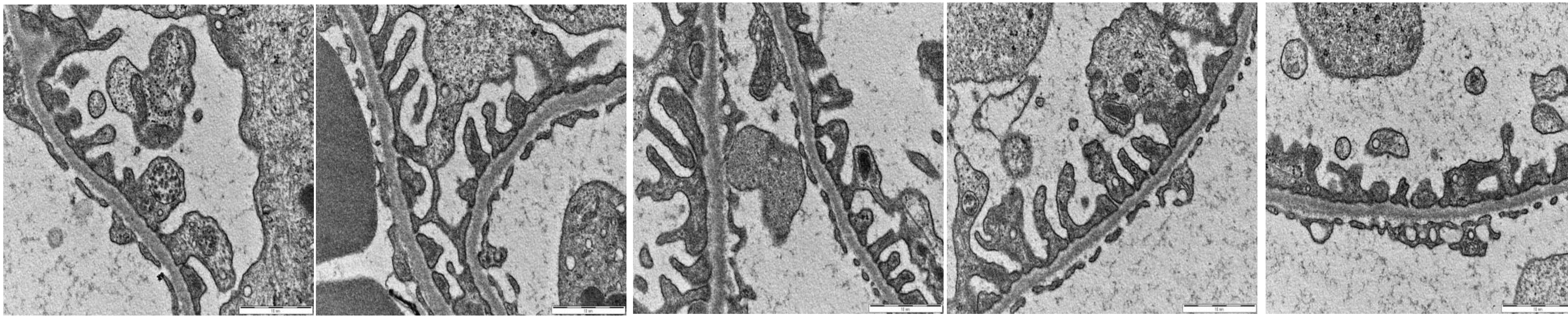

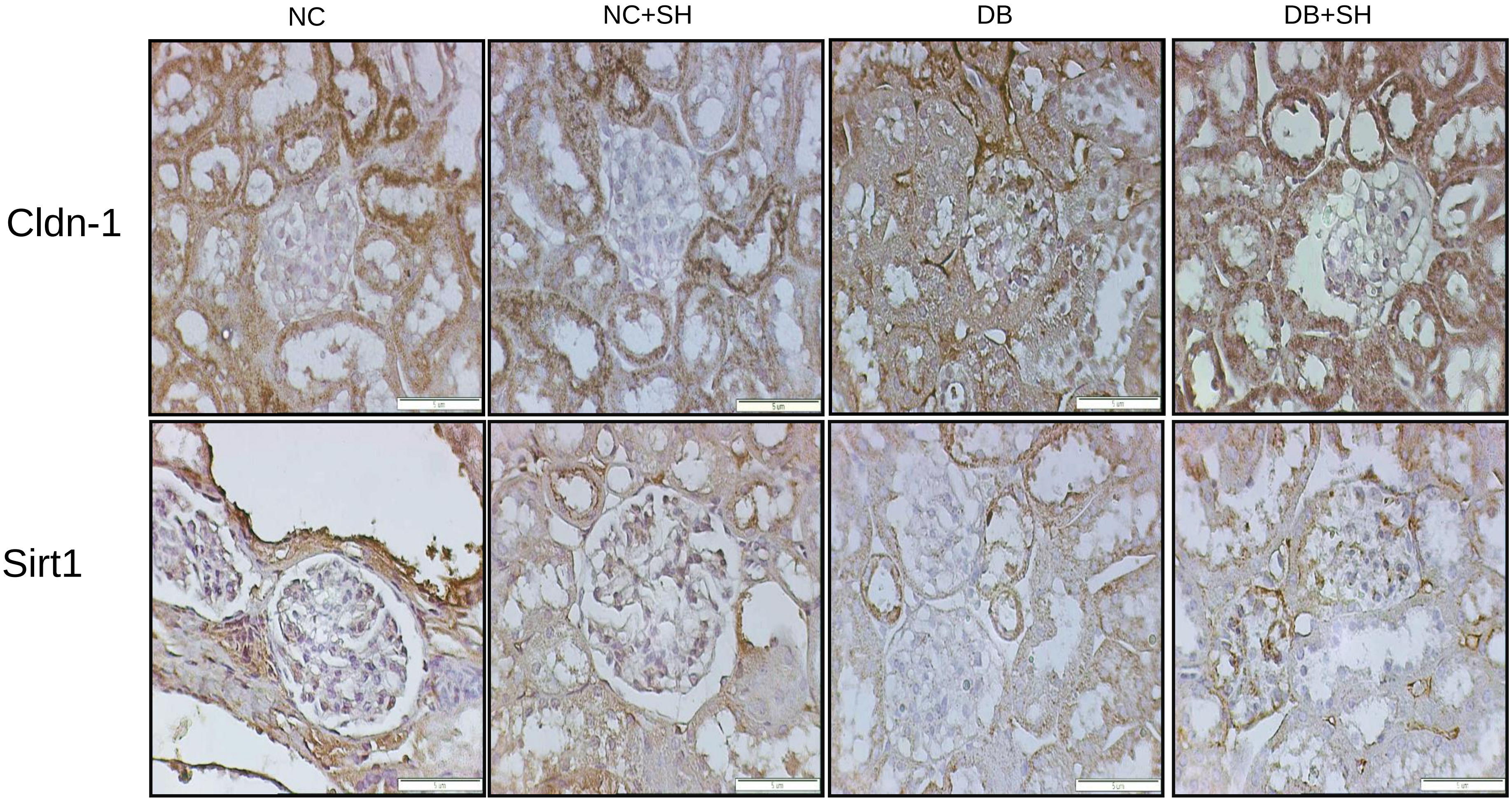
NC
NC+SH
DB
DB+SH
Cldn-1
Sirt1

LPS stimulated 5HT-2A expression in Raw264.7 cells (magnification, x 40)

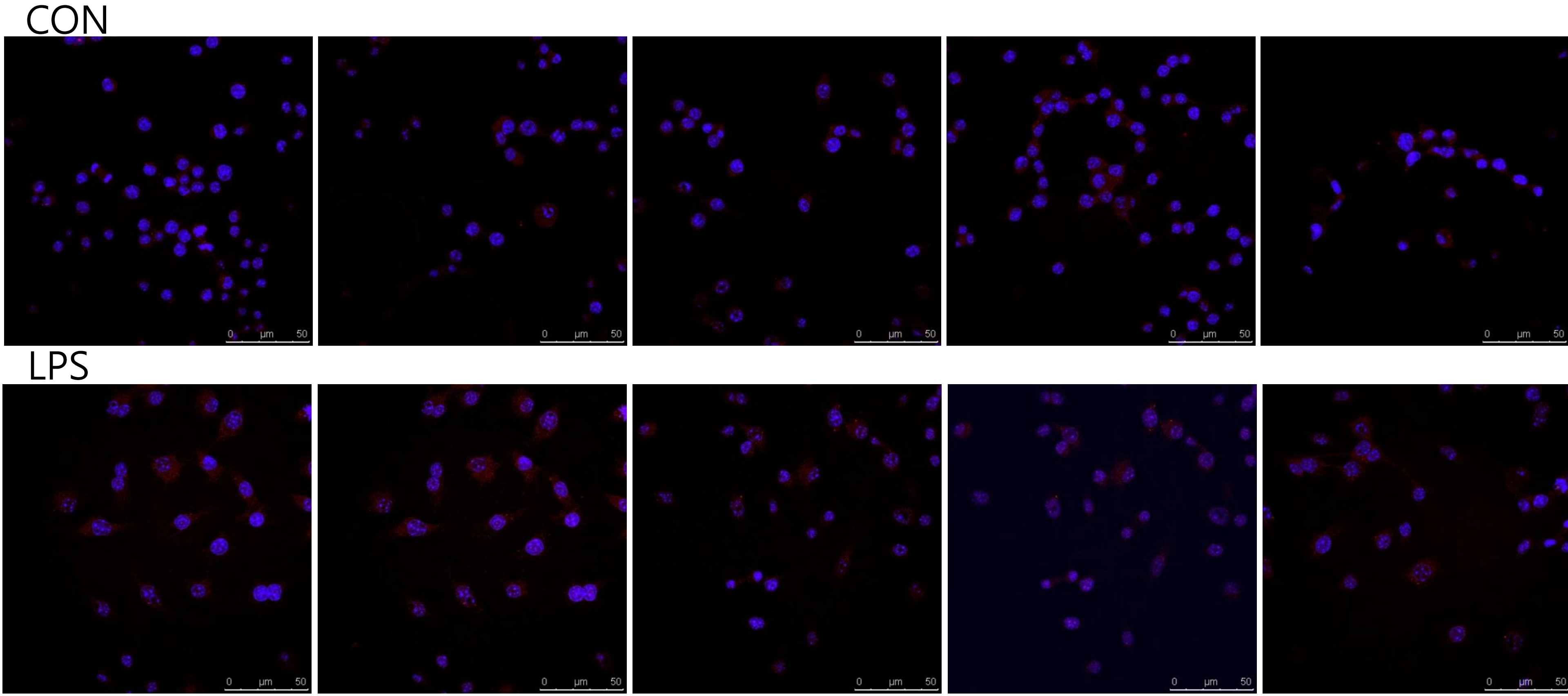

Blue: DAPI
Red: 5HT-2A

MacrophageMigration assay (magnification, x 100)

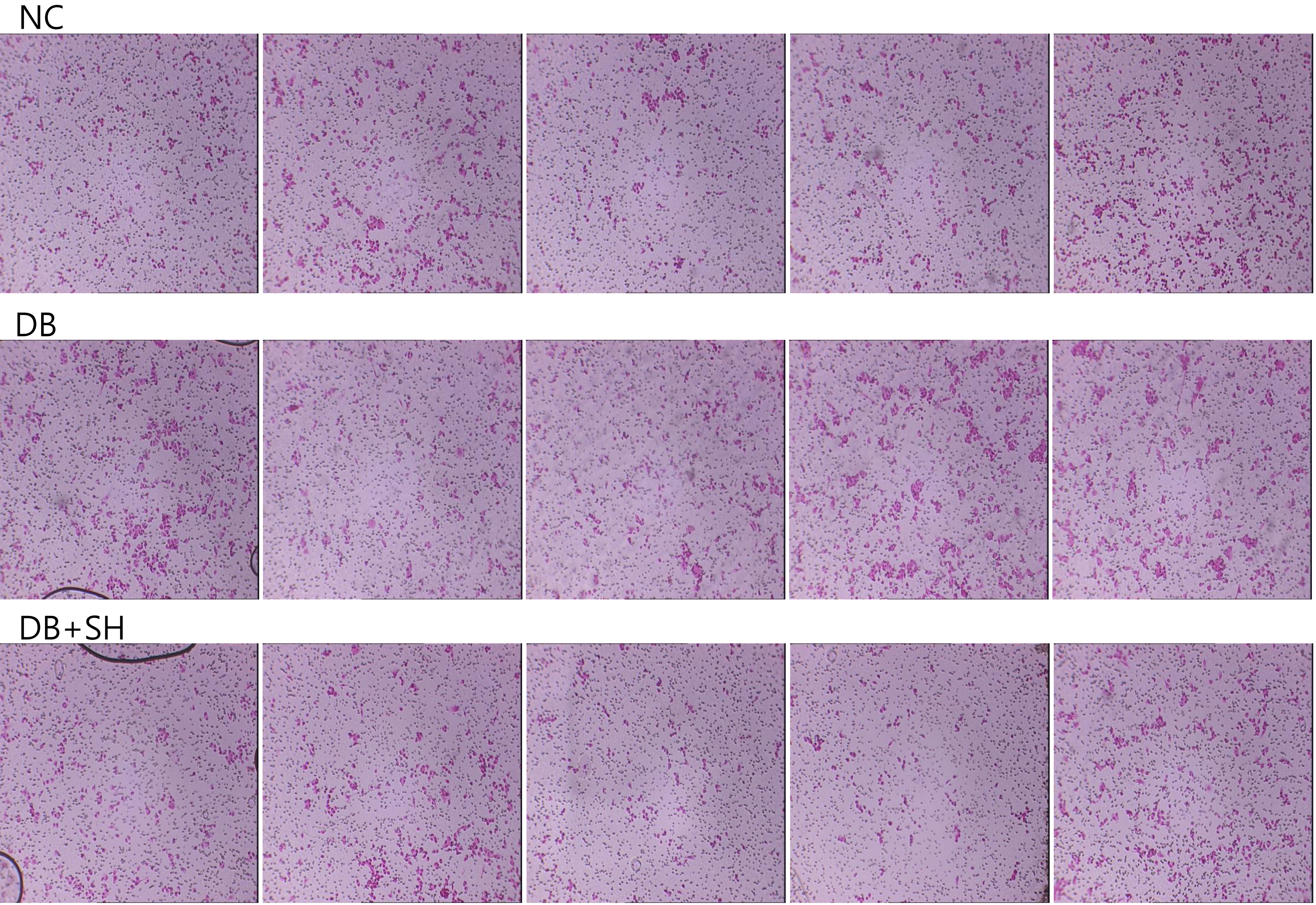

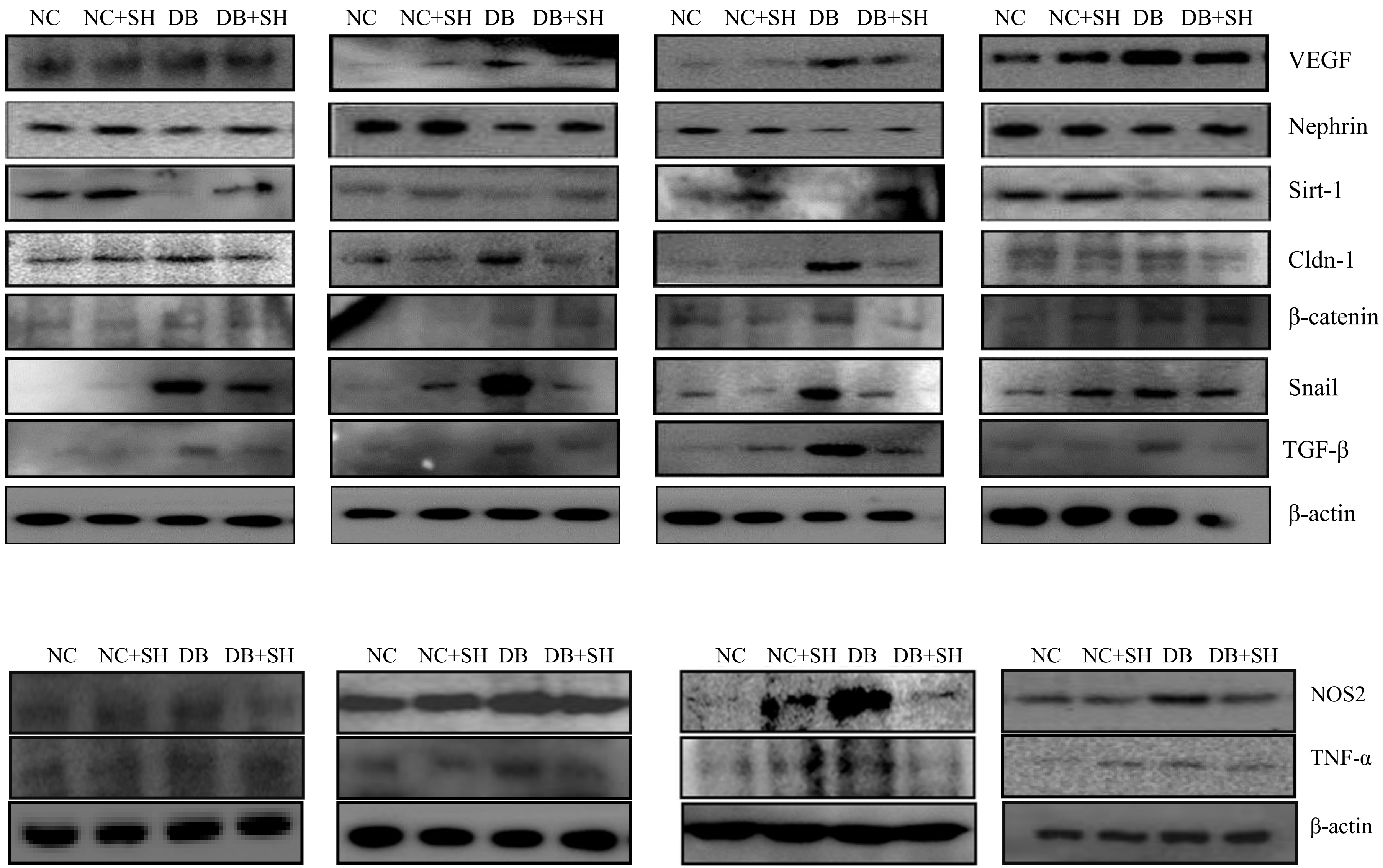
NC NC+SH DB DB+SH
NC NC+SH DB DB+SH
NC NC+SH DB DB+SH
NC NC+SH DB DB+SH
VEGF
Nephrin
Sirt-1
Cldn-1
β-catenin
Snail
TGF-β
β-actin
NC NC+SH DB DB+SH
NC NC+SH DB DB+SH
NC NC+SH DB DB+SH
NC NC+SH DB DB+SH
NOS2
TNF-α
β-actin

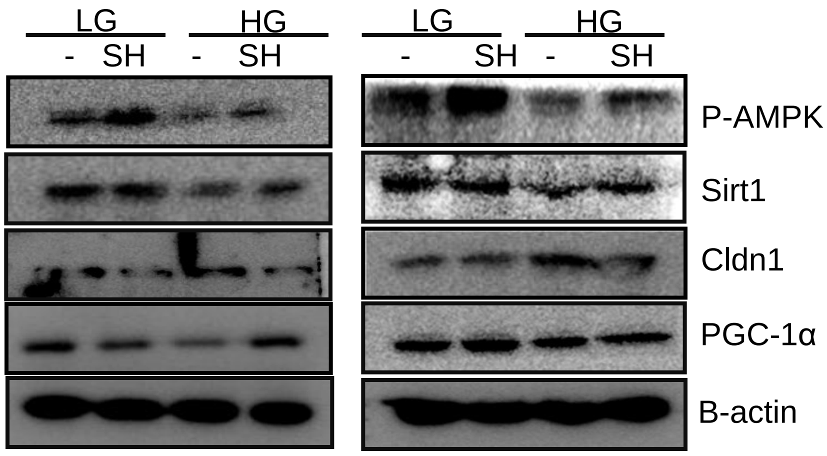
LG
HG
-
SH
P-AMPK
Sirt1
Cldn1
PGC-1α
B-actin

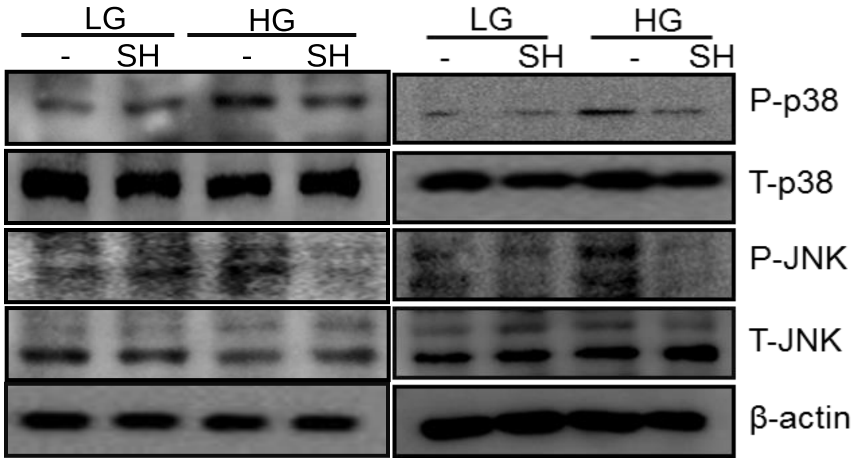
LG
HG
-
SH
P-p38
T-p38
P-JNK
T-JNK
β-actin

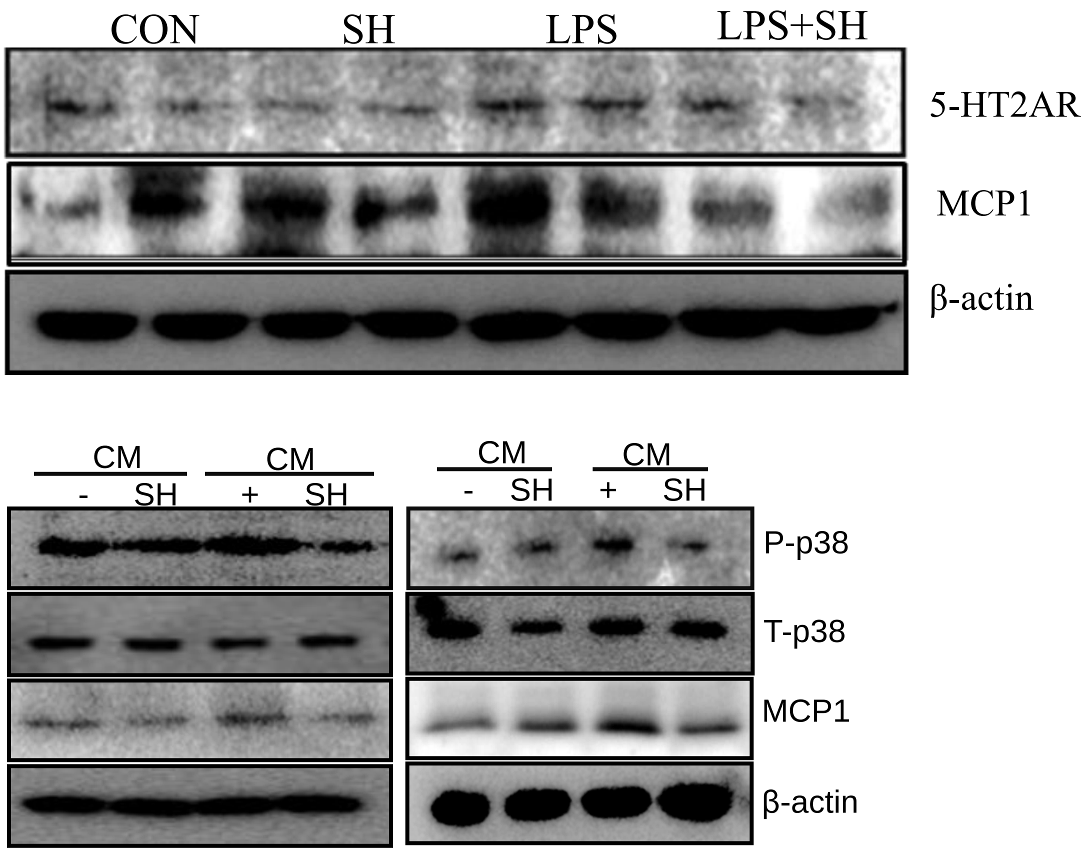
CON
SH
LPS
LPS+SH
5-HT2AR
MCP1
β-actin
CM
-
SH
CM
+
SH
CM
-
SH
CM
+
SH
P-p38
T-p38
MCP1
β-actin
